# Supervised Learning Classification Models for Prediction of Plant Virus Encoded RNA Silencing Suppressors

**DOI:** 10.1371/journal.pone.0097446

**Published:** 2014-05-14

**Authors:** Zeenia Jagga, Dinesh Gupta

**Affiliations:** Bioinformatics Facility, Structural and Computational Biology Group, International Centre for Genetic Engineering and Biotechnology, Aruna Asaf Ali Marg, New Delhi, India; CSIR-Institute of Microbial Technology, India

## Abstract

Viral encoded RNA silencing suppressor proteins interfere with the host RNA silencing machinery, facilitating viral infection by evading host immunity. In plant hosts, the viral proteins have several basic science implications and biotechnology applications. However *in silico* identification of these proteins is limited by their high sequence diversity. In this study we developed supervised learning based classification models for plant viral RNA silencing suppressor proteins in plant viruses. We developed four classifiers based on supervised learning algorithms: J48, Random Forest, LibSVM and Naïve Bayes algorithms, with enriched model learning by correlation based feature selection. Structural and physicochemical features calculated for experimentally verified primary protein sequences were used to train the classifiers. The training features include amino acid composition; auto correlation coefficients; composition, transition, and distribution of various physicochemical properties; and pseudo amino acid composition. Performance analysis of predictive models based on 10 fold cross-validation and independent data testing revealed that the Random Forest based model was the best and achieved 86.11% overall accuracy and 86.22% balanced accuracy with a remarkably high area under the Receivers Operating Characteristic curve of 0.95 to predict viral RNA silencing suppressor proteins. The prediction models for plant viral RNA silencing suppressors can potentially aid identification of novel viral RNA silencing suppressors, which will provide valuable insights into the mechanism of RNA silencing and could be further explored as potential targets for designing novel antiviral therapeutics. Also, the key subset of identified optimal features may help in determining compositional patterns in the viral proteins which are important determinants for RNA silencing suppressor activities. The best prediction model developed in the study is available as a freely accessible web server pVsupPred at http://bioinfo.icgeb.res.in/pvsup/.

## Introduction

RNA silencing is an evolutionary conserved sequence specific mechanism of post transcriptional gene silencing in eukaryotes which confers innate immunity against viruses [1]. In the evolutionary arms race, viruses have adopted various strategies to escape host immune systems. RNA silencing suppressors are the viral encoded proteins evolved with the capability to block host RNA silencing response [2], [3]. To disrupt RNA silencing machinery, virus encoded RNA silencing suppressors target several effectors of RNAi silencing pathway, such as viral RNA recognition, dicing, RISC assembly, RNA targeting and amplification [4], [5] e.g. P14 of *Pothos latent aureusvirus* and P38 of *Turnip crinkle virus* have been shown to inhibit the processing of dsRNA to siRNA [6], [7]. In another case, the P19 protein of *tombusviruses* prevents RNA silencing by siRNA sequestration through binding ds siRNA with a high affinity [8]. Other RNA silencing suppressors, such as the *Tomato aspermy cucumovirus* 2b protein or B2 of the insect-infecting *Flock House virus*, also bind ds siRNA in a size-specific manner [9], [10]. P0 of *Beet Western Yellow virus* directly interacts with AGO1 promoting its degradation [11]. P6 protein of *Cauliflower Mosaic virus* directly interacting with dsRNA binding protein 4 required for DCL4 functioning [12]. The viral proteins identified mostly in plants and some insects or mammalian viruses, show diversity within and across the taxonomic kingdoms [13].

Along with the suppression activity, these viral proteins have multifunctional and indispensable role in viruses as coat proteins, movement proteins, replicases, proteases, helper components for viral transmission and transcription regulation [14]. This makes exploration of these proteins difficult, as inactivation of these proteins risks viability of a given virus in virus-targeted RNA silencing mediated gene knock down experiments. Experimental screening is performed by agrobacterium mediated *in planta* assay systems based on reversal of silencing and enhancement of rolling circle replication of geminivirus replicon [15]. Non availability of quick screening method is a major limitation for identification of viral suppressors of RNA silencing. Hence, not many such proteins have been reported till date. Thus, apriori knowledge about endowment of RNA silencing suppression capability of a new pathogenic viral protein would guide the future drug/vaccine strategies against the new viruses [16], [17], other applications like molecular biofarming [18], si/miRNA based antiviral gene therapy [19] and as biosensors for miRNA [20].

Identification of RNA silencing suppressor activity in viral proteins has important implications in studying various facets of viral infection and pathogenesis and understanding the mechanism and function of RNA silencing machinery [21]. Since, the experimentally validated viral suppressors exhibit considerable diversity in its primary sequences, structures, origins, mode of suppressor action and evolution [4], [5]. Owing to its inherent diversity and complexity, identification of novel RNA silencing suppressor sequences in virus proteomes using conventional similarity based bioinformatics approaches is difficult. Existing approaches to identify RNA silencing suppressor activities investigate the presence of RNA binding motif and/or motifs responsible for binding to the components of silencing machinery – for example GW/WG motif for Argonaute binding [16]. However, the presence of these motifs *per se* is not a confirmatory evidence to annotate a viral protein as viral RNA silencing suppressor.

Machine learning algorithms have been extensively implemented to detect cryptic patterns in disparate biological domains. In particular, supervised machine learning algorithms, have been very effective to investigate biological classification problems [22], [23]. The generated classification models have served as valuable tools to further predict the new cases of the same class. Absence of any computational algorithm to identify viral RNA silencing suppressor sequences motivated us to undertake this study. We have developed prediction models for viral RNA silencing suppressors protein sequences and evaluated these by implementing four supervised machine-learning algorithms, namely- Naïve Bayes, J48, Random Forest and LibSVM. Feature vector profiles based on various structural and physicochemical features of experimentally verified viral suppressor proteins of RNA silencing were used for algorithm trainings. Our present study marks an effort, to predict unexplored viral RNA silencing suppressors, which could be potential targets for designing novel antiviral therapeutics, and enhance our understanding of RNA silencing. In this direction, we found that among the four machine learning techniques implemented by us, the classifier model based on Random Forest algorithm was the best prediction classifier.

## Materials and Methods

**Figure 1:**
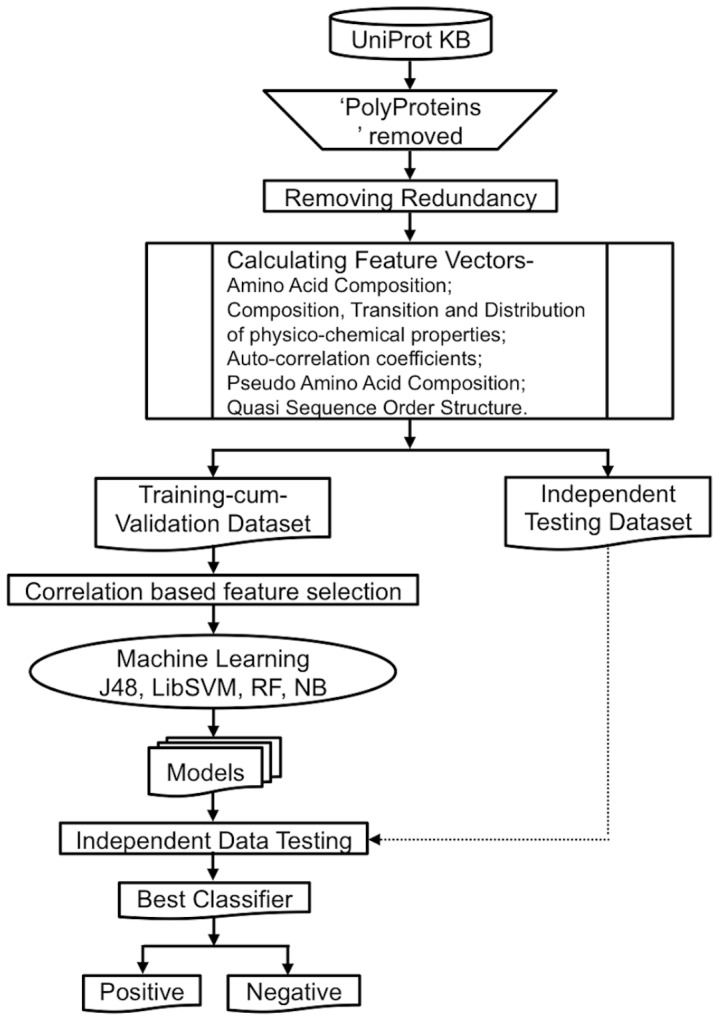
Methodology flow. Framework of the computational method used for development of predictions models for plant virus encoded RNA silencing suppressors.

The general flow of methodology adopted in this study for data mining, feature calculation, feature selection, analysis, model building and validation has been schematically represented in Figure 1.

### Data Mining

We generated the training dataset by obtaining protein sequences by searching UniProtKB database release 2013_06 [24] using combination of different query terms and logical variables. For example, we obtained positive dataset (X) of ‘viral protein sequences which have Kingdom Plantae as hosts and are viral suppressor for RNA silencing’ using the query: ‘taxonomy:Viruses reviewed:yes fragment:no host:33090 keyword:KW-0941’. Similarly, we obtained the negative dataset (X′) as ‘viral protein sequences which have natural host plant kingdom hosts but are not viral suppressor for RNA silencing’ by using the query ‘taxonomy:Viruses reviewed:yes fragment:no host:33090 NOT keyword:KW-0941’. We limited our selection criterion to include only experimentally verified and complete sequences. We removed the sequences annotated as ‘polyprotein’ in sequence headers information of fasta formatted positive and negative datasets sequences downloaded from UniProtKB. Further, we manually analyzed the positive dataset polyprotein sequences to process fragments into positive (A) and negative (B) polyprotein subsets. We discarded the polyprotein sequences from the negative dataset (Y′). Hence, we obtained the final positive dataset Z, as X−Y+A, and similarly the final negative dataset ‘Z’ as X′−Y′+B. We retrieved the final positive dataset (Dataset S1) and final negative dataset (Dataset S2) in fasta format by batch retrieval system of the UniProt database.

### PSI-BLAST similarity based Search

We used standalone PSI-BLAST version 2.2.27+ with a threshold E-value 0.001, number of iterations three over final positive dataset i.e. 208 viral RNA suppressor sequences by Leave One Out Cross-Validation (LOO-CV). Each sequence of the dataset was iterated as the query sequence once against rest sequences as reference database.

### Preparing Non-Redundant Dataset

**Figure 2:**
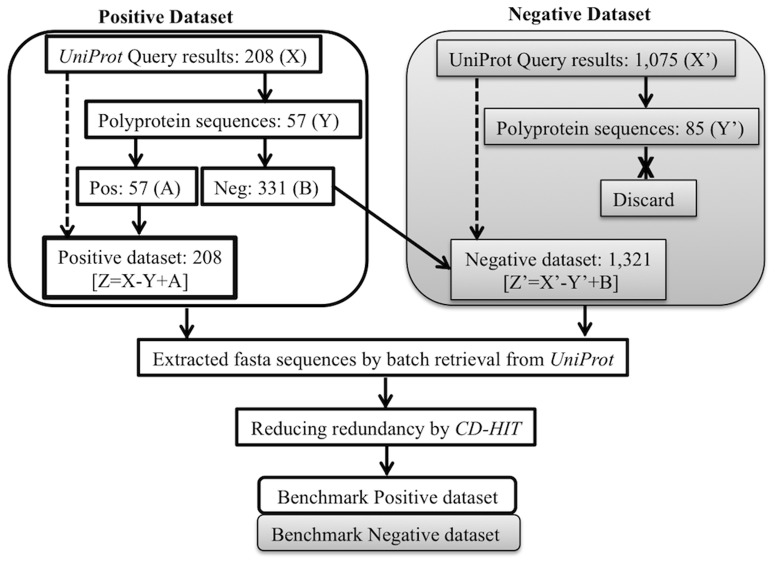
Schematic representation of the dataset generation and filtration steps. The figure shows flowchart for the steps followed for sequence collection, filtering and redundancy removal for training dataset generation.

To prepare a non redundant dataset to avoid over-fitting problem, we used CD-HIT (Cluster Database at High Identity with Tolerance) version 4.5.7 [25], [26]. CD-HIT works on 'the longest sequence first' list removal algorithm for removing redundant sequences from the dataset. We utilized redundancy threshold parameter to generate datasets of redundancy 90%, 70% and 40%. As dataset removing redundancy to 40% reduced dataset size to less than half (Table 1). Thereby we proceeded to calculate feature vectors for two redundancy levels −90% and 70% datasets. Figure 2 summarizes the dataset generation and filtering methodology followed in the study.

**Table 1:** Number of protein sequences after removing redundant proteins at thresholds of 90%, 70% and 40% using CD-HIT.

Redundancy cut off	Positive Dataset (208)	Negative Dataset (1321)
**90%**	142	949
**70%**	118	815
**40%**	66	555

### Calculation of feature vectors

Performing feature selection is a useful measure to avoid over-fitting of the models, which also improves model performance, cost-effectiveness of models, and to understand which features would be predictive in nature [27], [28]. In this study, we calculated the feature vectors from the dataset sequences using propy 1.0 [29]. Propy is a python based package which calculates comprehensive structural and physicochemical features from protein sequences, in five feature groups – Amino Acid Composition (AAC), Composition Transition and Distribution (CTD) of various physico-chemical properties, Auto-correlation Coefficients (AC), Pseudo Amino Acid Composition (PAAC) and Qausi Sequence Order structure (QSOD) [29]. AAC includes percentage amino acid composition of each amino acid, i.e. 20 feature vectors along with dipeptide composition of protein represented by 400 feature vectors. AC incorporates three subgroups, i.e. normalized Moreau-Broto autocorrelation, Moran autocorrelation and Geary autocorrelation with feature vector dimension of 240 each, describing correlation based on specific structural or physicochemical properties. CTD group represent amino acid distribution pattern of the following physicochemical properties: hydrophobicity, normalized van der Waals volume, polarity, charge, secondary structure, solvent accessibility with feature vector dimension of 147. QSOD has two sequence-order sets namely, quasi-sequence-order with 100 feature vectors and sequence-order-coupling number with 90 feature vectors derived from Schneider–Wrede physicochemical and Grantham chemical distance matrix. PAAC consists of 2 subgroups, type I PAAC and type II PAAC (i.e. amphiphilic PAAC), with feature vector dimension of 60. Use of features based on Chou's pseudo-amino acid compositions have been widely used for development of machine learning models for predicting protein structural and functional classes [30]–[32]. We converted the propy outputs in dictionary format to comma separated value format with IDs in rows and feature vectors in corresponding columns. Additionally, we added a class label ‘Outcome’ as ‘active’ for positive dataset and as ‘inactive’ for negative dataset. Before calculating descriptors, propy also validates the protein sequences by Procheck python module to eliminate protein sequences having non-conventional amino acids. Further, we performed a random-stratified splitting of benchmark dataset to generate training-cum-validation dataset (80%) and independent testing dataset (20%).

### Machine Learning

Waikato Environment for Knowledge Analysis (WEKA) version 3.6.10 was used for the data pre-processing, feature selection and classification experiments [33]. WEKA is a popular suite for various machine learning algorithms, having various tools for data pre-processing, classification, regression, clustering, association rules and visualization.

#### Data Pre-processing

The testing and training dataset in comma separated value (CSV) format were converted to attribute-relation file format (ARFF) files using weka.core.converters, to convert input files to WEKA readable format.

#### Feature Selection

To reduce the feature space dimensions, we combined the filter method “Correlation based feature selection” with “Best First search” strategy. Correlation based feature selection is a multivariate analysis to determines the feature subset that are highly correlated with the class, yet uncorrelated with each other [34]. “InfoGainAttributeEval” with “Ranker” strategy was implemented to determine contribution of each feature vector towards class label. We performed optimization of feature vector space only upon training-cum-validation datasets.

#### Algorithms for Classification

In the present study, four different state-of-art supervised machine learning algorithms namely J48, LibSVM, Naïve Bayes and Random Forest are used for model generation and comparison. J48 implements C4.5 decision tree learning algorithm [35]. LibSVM is a library of Support Vector Machines (SVM) implemented as a wrapper within WEKA [36], [37]. SVM algorithm generates a hyperplane in the feature space with maximum margin distinguishing positive instances from negative [38]. Naïve Bayes is a classifier based on Bayes theorem and assumes that evidence based on attributes which are statistically independent [39]. Random Forest is an ensemble classifier based on independent decision trees [40].

Initially we employed standard error base classifiers on the training data. As the dataset is unbalanced, and the base classifiers assume equal weighting of classes, we trained the models with cost sensitive learning algorithms optimizing mis-classification costs [41]. Cost sensitive meta-learning algorithm introduces cost sensitivity to its base classifier by two ways. This is achieved either by reweighting the training instances according to the cost assigned to each class or using minimum expected misclassification cost for predicting the class [42]. We used the former strategy by setting the parameter “*Minimize Expected Cost*” as false in the cost sensitive classifier.

#### Training-cum-validation

We developed the prediction models by training the chosen classifiers on the optimized features from feature selection and validating by 10 fold cross validation technique. We added arbitrary costs on the false negative rates to overcome class imbalance problem in the dataset, with the costs starting from 2 such that false positive rate would not exceed threshold of 20%. Further, we compared the model performances by standard statistical measures.

#### Independent dataset test

It is recommended to perform independent data testing to exclude the “memory” effect or bias in the predictive modelling [31]. In this study, we re-evaluated the best predictive models by 10 fold cross validation study on independent dataset. We analyzed and compared the performances of the proposed models using standard statistical measures.

### Classifier Evaluation

We used different evaluation metrics normally recommended for evaluating the classifier's performances- accuracy, sensitivity or recall, specificity, Balanced Classification Rate (BCR), F-value, Matthews Correlation Coefficient (MCC) and Area Under Recievers Operating Characteristic (auROC) curve [43], [44]. Accuracy provides the overall effectiveness of the classifier (1). Sensitivity or Recall determines classifier effectiveness to identify positive class labels (2). Specificity calculates the classier effectiveness to identify negative class labels (3). BCR, F-measure and MCC are the standard evaluation metric, in the case of class imbalance. BCR, also called as balanced accuracy, is the average of sensitivity and specificity (4). F measure combines recall and precision by harmonic mean (5). MCC value ranges from 0 to 1 where 1 is the perfect prediction and 0 is random prediction (6). Mathematical representation of these expressions is given below, where TP is the number of True Positive, TN is the number of True Negatives, FN is the number of False Negatives, and FP is the number of False Positives for a prediction method. Receiver Operating Characteristic (ROC) curve is a 2 dimensional graphical plot of FP rate on X-axis vs. TP rate on Y axis. The auROC provides a single measure in the case of comparing performance of several classifiers. 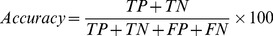
(1)

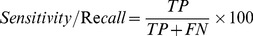
(2)

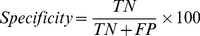
(3)

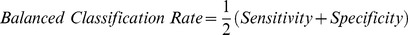
(4)

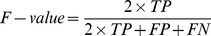
(5)

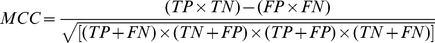
(6)


## Results and Discussion

### PSI-BLAST similarity based Search

Similarity based searches with annotated datasets is the first step for functional annotation of novel protein sequences. PSI-BLAST is a remote homology similarity and preferred algorithm for functional annotation projects. We employed PSI-BLAST, by leave-one out strategy on final 208 proteins positive dataset and obtained no significant hits for 20 sequences. This reinforced the need to develop alternative prediction tools for viral suppressor proteins.

### Feature Selection

The python tool propy yielded 1537 feature vectors from five different feature groups i.e. AAC, AC, CTD, PAAC, QSOD for each instance. The dataset was then divided into training-cum-validation and test dataset (Table 2). Feature vector optimization performed on training-cum-validation data by correlation based feature selection reduced the feature vectors from 1537 to 73 in dataset of 90% redundancy levels and 77 in dataset with 70% redundancy levels. To have an understanding of which feature vectors contributed to the optimal subset, we investigated the distribution of each type of feature for feature group category (Table 3). Intriguingly, we observed that the optimal feature subset had representation from all feature groups except QSOD. We have provided the description of the features calculated in each feature group category, the selected optimal features and the information gain values as Dataset S3.

**Table 2:** Number of positive and negative instances in testing and training dataset.

Redundancy (%)	Class	Training Data	Testing Data
**90**	Positive	107	26
**90**	Negative	739	185
**70**	Positive	89	22
**70**	Negative	633	158

**Table 3:** Feature distribution of optimal feature subset generated by correlation based feature selection.

Feature Group	Feature	Feature vectors calculated	Feature vectors selected	
Redundancy level			90%	70%
**AAC**	Amino acid composition	20	2	3
**AAC**	Dipeptide composition	400	39	43
**CTD**	Composition	21	1	1
**CTD**	Transition	21	1	NA
**CTD**	Distribution	105	10	10
**AC**	Geary autocorrelation	240	6	6
**AC**	Moran autocorrelation	240	5	3
**AC**	Normalized Moreau-Broto autocorrelation	240	1	NA
**PAAC**	Type 1 pseudo-amino acid composition	20	5	5
**PAAC**	Type 2 pseudo-amino acid composition	40	3	5
**QSOD**	Quasi-sequence-order desciptors	100	NA	1
**QSOD**	Sequence-order-coupling number	90	NA	NA
		1537	73	77

### Training-cum-validation

To come up with the best prediction model, we compared the prediction performance of the four machine learning approaches, namely LibSVM, Random Forest, J48 and Naïve Bayes. We generated seven training-cum-validation dataset and independent testing dataset for 90% and 70% redundancy i.e. AAC, AC, CTD, PAAC, QSOD, all descriptors and optimized feature set. We performed the initial experiments with standard error base classifiers and later standardized with meta-learner cost sensitive classifiers. We introduced misclassification cost on false negatives and augmented to a threshold of 20% for FP rate. Thereby, we generated a large number of models with different cost settings. As expected we observed that introducing cost for FN, decreased the number of FN and increased FP (data not shown). Hence, cost sensitive classifiers lead to more robust models as compared to standard classifiers. We generated all the training models by 10 fold cross validations, the detailed statistical evaluation for 57 generated models is given in Dataset S4. We observed that prediction models generated with dataset redundancy 70% performed better than prediction models generated from dataset redundancy 90%. Within training models generated from dataset with redundancy 70%, prediction models with optimized feature vectors performed better than classifiers generated by individual feature class. Henceforth, we will mainly discuss the training models generated with 70% redundancy threshold, using 77 feature vectors optimized by correlation based feature selection.

Table 4 shows the misclassification cost, accuracy, sensitivity, specificity, BCR, F-value, MCC and auROCs of the best prediction models generated with dataset of 70% redundancy. We observed that LibSVM required minimum misclassification cost settings of 18 and Naïve Bayes required a maximum of 4200. All the classifiers had their accuracies approximately around 80%. The auROC values determined that the models were predictive in nature and not random in their performance. Based on the highest value of calculated statistical evaluators, Random Forest trained prediction model performed the best amongst the four implemented machine learning techniques.

**Table 4:** Statistics of best predictive models generated by 10 fold cross-validation of training dataset.

Classifier*	CSC J48	CSC LibSVM	CSC Naïve Bayes	CSC Random Forest
**Misclassification Cost**	42	18	4200	55.4
**Accuracy**	79.50	78.25	78.25	**80.61**
**Sensitivity**	73.03	62.92	65.17	**80.90**
**Specificity**	80.41	80.41	80.09	**80.57**
**BCR^$^**	76.72	71.67	72.63	**80.73**
**F-value**	0.47	0.42	0.42	**0.51**
**MCC^∧^**	0.40	0.33	0.34	**0.46**
**auROC^#^**	0.79	0.72	0.79	**0.91**

*CSC denotes Cost Sensitive Classifier; BCR^$^ is Balanced Classification Rate; MCC^∧^ is Matthews Correlation Coefficient; auROC^#^ is area under Receiver Operating characteristic curve. Highest numerical value in each row is highlighted as bold.

### Independent-dataset Testing

We performed an independent data testing to further assess the performances of the best prediction models obtained from cross validation studies on unseen data. The evaluation of comparative performance of the classifiers has done using standard statistical measures i.e. accuracy, sensitivity, specificity, BCR, F-measure, and auROC plot. Table 5 summarizes the results from these statistical measures. We found the performance of the models with independent dataset in terms of accuracy and auROC in coherence with the cross validation results.

**Table 5:** Summary of statistical measures of the best classifiers on re-evaluation with independent dataset.

Classifier*	CSC J48	CSC LibSVM	CSC Naïve Bayes	CSC Random Forest
**Accuracy**	85.0	78.33	78.33	**86.11**
**Sensitivity**	81.82	77.27	68.18	**86.36**
**Specificity**	85.44	78.48	79.75	**86.08**
**BCR^$^**	83.63	77.88	73.96	**86.22**
**F-value**	0.57	0.47	0.43	**0.60**
**MCC^∧^**	0.53	0.41	0.36	**0.57**
**auROC^#^**	0.84	0.78	0.83	**0.95**

*CSC denotes Cost Sensitive classifier; BCR^$^ is Balanced Classification Rate; MCC^∧^ is Matthews Correlation Coefficient; auROC^#^ is area under Receiver Operating Characteristic (ROC) curve. Highest numerical value in each row is highlighted as bold letters.

**Figure 3:**
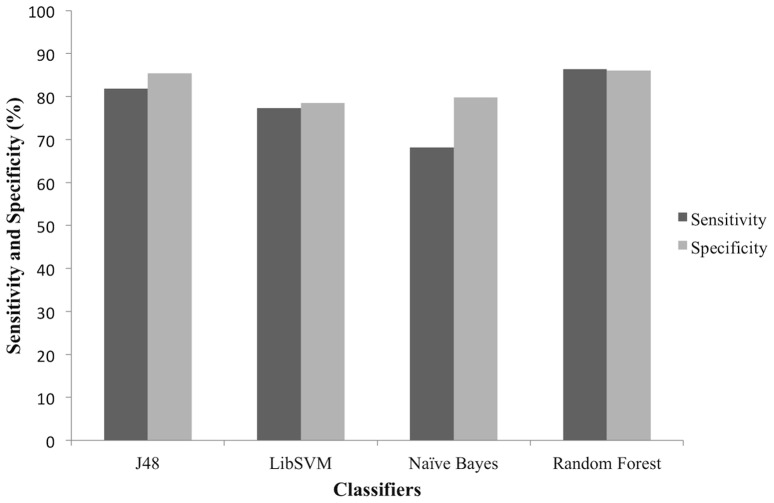
Sensitivity and Specificity plot. The plot compares sensitivity and specificity of the developed predictive models, to determine effective classifier for identifying positive and negative instances. Random Forest classifier has the highest sensitivity and specificity values as compared to J48, LibSVM and Naïve Bayes.

Sensitivity-specificity plots help identification of the best model which correctly classifies positive and negative labelled instances (Figure 3). Specificity of all the models was in the range of 78–86%, however the prediction model sensitivities varied in a wide range from a minimum of 68.19% for Naïve Bayes, 77.27% for LibSVM and 81.82% for J48 to highest 86.36% for Random Forest algorithm based model.

LibSVM and Naïve Bayes had minimum predictive accuracies of 78.33%, with 85% of J48 classifiers and maximum accuracy 86.11% for Random Forest (Table 5). Owing to the class imbalance problem in the training dataset, we also calculated BCR, F-measure and MCC to precisely evaluate model effectiveness. F-value of the models was in the range of 0.43–0.60. Although the models may not have high F value, the remarkably high sensitivity or recall (92.31%) of the model based on Random Forest algorithm is noteworthy.

**Figure 4:**
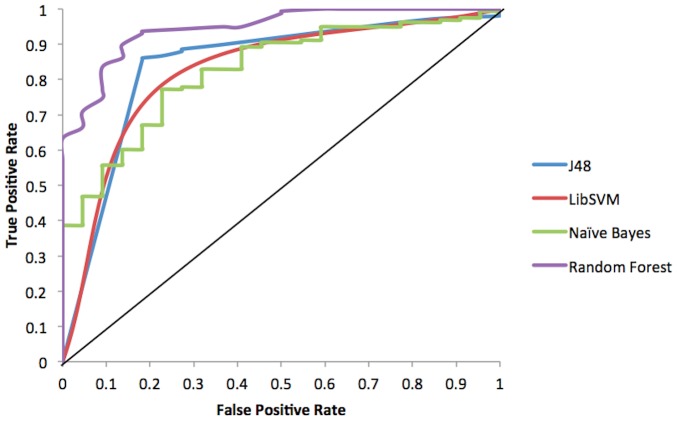
ROC curves for the predictive performance of different cost sensitive classifiers. ROC plot depicts a relative trade-off between true positive rate and false positive rate of the predictions. The diagonal value represents a completely random guess. The corresponding scalar values of area under curve are given as auROC in table 4.

ROC analysis is an established approach for classifier evaluation in the Machine learning approaches, as it shows the trade-off between true positive rate and false positive rate. The ROC plot of the classifiers in Figure 4 and auROC values (Table 5) suggest that the performance of the Random Forest was the best, followed by J48, Naïve Bayes and LibSVM. The observed auROC values (Table 5) were significantly higher than threshold of 0.5 i.e. random guess as prediction. Thus, ROC analysis assured for the optimal and robust performance of Random Forest.

Random Forest model ranks above the J48, Naïve Bayes and LibSVM, as evident from the calculated statistical evaluators. Hence, the Random Forest algorithm based model is the most efficient model to classify viral sequences as viral RNA silencing suppressors and silencing non suppressors with an accuracy of 86.22%, BCR of 86.22%, MCC of 0.57 and remarkably high auROC of 0.95.

During the preparation of this manuscript, a new plant virus encoded viral supressor was included in UniProtKB. The protein, CLINK_BBTVA is encoded in Banana Bunchy top virus, annotated as viral suppresor of RNA silencing. The sequence when tested as a new blind independent dataset sequence for the Random Forest algorithm based prediction models, is correctly predicted as a suppressor of RNA silencing. The result further confirms the reliability of the Random Forest based classifier.

### Implementation

**Figure 5:**
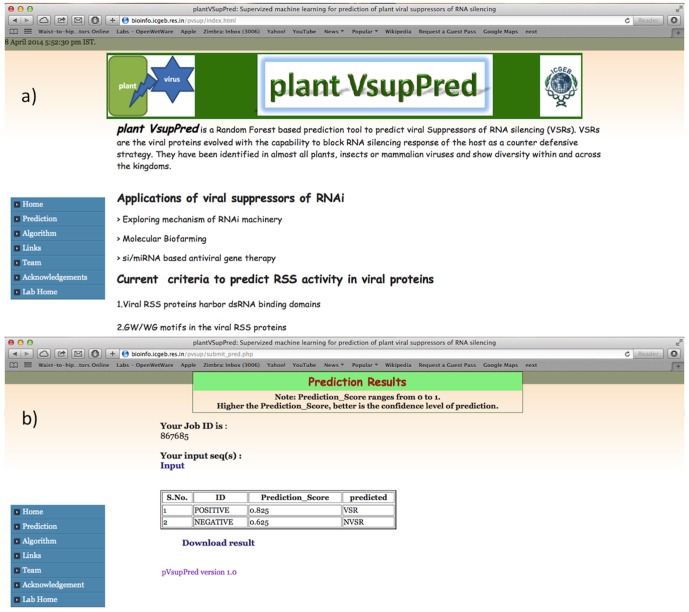
Snapshot of pVsupPred web server. The web server predicts viral suppressor of RNA silencing based on Random Forest classifier generated in this study. Figure 5a is snapshot of prediction page of pVsupPred webserver. The webserver accepts FASTA formatted protein sequences. Figure 5b is snapshot of sample output of pVsupPred web server. The prediction threshold range of the developed method is 0 to 1.0.

The best performing Random Forest based prediction model is implemented as a freely accessible web server pVsupPred (Figure 5, http://bioinfo.icgeb.res.in/pvsup/). Scripting is done in HTML, PHP, PERL and shell to develop the user friendly interface. The server accepts input protein sequences in FASTA format. The VsupPred web server results are generated in simple tabular format which includes sequence ID, prediction score and decision of the model regarding the sequence. The higher prediction scores indicate better confidence level of prediction.

### Perspective

pVsupPred can be potentially used for screening RNA silencing suppressors in viral proteomes. Since the development and affordability of next generation sequencing, there lies a huge gap between sequencing and annotation projects. pVsupPred is one such endeavor which can be helpful in the annotation projects of genomic data of viruses. We anticipate that our implemented methodology would be useful for experimental biologists and research community as a whole. In future, with availability of additional experimentally validated viral suppressor's sequences, performance of the models could be further improved.

## Conclusion

In this study, we employed a empirical approach to develop a prediction method for plant viral RNA silencing suppressors by developing statistical prediction models based on features of the experimentally verified viral suppressor protein sequences. We have generated classification models with four supervised learning classifiers i.e. Naïve Bayes, Random Forest, J48 and LibSVM. Feature selection by correlation based feature selection increased the robustness of the models and helped identification of optimal features which reflects compositional patterns in the viral proteins responsible for RNA silencing suppressor activities. Further introducing meta-learning by cost sensitive classifiers led to enhanced and robust performance of models. Random Forest predictive model achieved the best performance when compared with Naïve Bayes, J48 and LibSVM models. We expect that these prediction models can aid screening of plant viral ORFs for potential suppressor activity for RNA silencing. Currently, the best prediction model developed in this study is available as web server pVsupPred. To the best of our knowledge, this is the first exclusive prediction method for plant viral suppressors of host RNA silencing.

## Supporting Information

Dataset S1 **Positive Benchmark dataset.** This consists of 208 viral RNA silencing suppressor protein sequences in fasta format. The file can be viewed using any text editor like wordpad or Notepad.
(FASTA)

Dataset S2 **Negative Benchmark dataset.** This consists of 1321 viral protein sequences which are RNA silencing suppressor in fasta format. The file can be viewed using any text editor like wordpad or Notepad.
(FASTA)

Dataset S3 **Feature Vectors.** This contains the information about the description of total feature vectors calculates and the ones selected by feature selection in an excel worksheet.
(XLSX)

Dataset S4 **Statistical Evaluation of Classifiers.** This contains the information about the statistical evaluators and parameter details used for generating classifiers by 10 fold cross-validation and re-evaluation of the classifiers by independent dataset at redundancy level of 90% and 70%.
(XLSX)
